# Parasitoids of *Delia planipalpis* (Meigen) and *Delia platura* (Stein) (Diptera, Anthomyiidae) in Mexico

**DOI:** 10.3897/zookeys.1046.64405

**Published:** 2021-06-21

**Authors:** Paulina Nava-Ruiz, Ricardo Meraz-Álvarez, Jorge Valdez-Carrasco, Onésimo Chávez-López, Néstor Bautista-Martínez

**Affiliations:** 1 Campo Experimental de Delicias CIRNOC-INIFAP, Km 2 carretera Delicias-Rosales, Delicias C.P. 33000, Chihuahua, México Campo Experimental de Delicias CIRNOC-INIFAP Delicias Mexico; 2 Colegio de Postgraduados, Posgrado en Fitosanidad-Entomología y Acarología, Carretera México-Texcoco Km 36.5, Montecillo, Texcoco 56230, Estado de México, México Colegio de Postgraduados Texcoco Mexico; 3 Iniciativa Privada, Texcoco, México Iniciativa Privada Texcoco Mexico

**Keywords:** Biological control, crucifers, Hymenoptera, root maggots, soil pests, Staphylinidae

## Abstract

Among the insect pests that affect crucifer crops in Mexico are *Delia planipalpis* (Meigen) and *D. platura* (Stein). They are a threat to the production of these vegetables since the damage they cause directly and indirectly affects yield, quality, and commercialization of these crops. Nevertheless, the existence of natural enemies of these dipterans is still unknown. It is fundamental to determine which parasitoids or predators can be considered possible biological control agents in an integrated pest management program.

The sampling sites were located in Guanajuato, Puebla, and the State of Mexico, where plants of Brassica oleracea L. var. italica Plenk and *capitata* L., *B. napus* L., and *Raphanus sativus* L. infested with *Delia* spp. were selected. The symptoms observed were wilting, yellowish, flaccid leaves and individuals less developed than the rest of the crop. These plants were extracted with their root and the surrounding soil. Also, wild crucifers were collected, such as *Raphanus raphanistrum* L., *Brassica campestris* L., and *Sisymbrium irio* L. The first records of *Aphaereta pallipes* Say (Hymenoptera, Braconidae), *Trybliographa rapae* (Westwood) (Hymenoptera, Figitidae), and *Aleochara bimaculata* Gravenhorst (Coleoptera, Staphylinidae) are reported parasitizing the puparia of these anthomyiid flies on cultivated and wild crucifers. This represents only a starting point for the continuous study of these parasitoids, which is needed to consider them useful for the biological control of *D. planipalpis* and *D. platura*.

## Introduction

Some species of the genus *Delia* (Robineau-Desvoidy) (Diptera, Anthomyiidae), commonly called root maggots, attack economically important crops of the family Brassicaceae (Brooks 1951; Finch 1989). These insects cause large economic losses to agriculture in temperate regions between latitudes of 30°N and 60°N (De Wilde 1947; Dixon et al. 2014). One example is the cabbage root fly, *Delia radicum* L., which is distributed in Europe (Belgium, Denmark, and Spain), North America (Canada and the United States), Asia (China, Turkey, and Israel), and Africa (Algeria and Morocco) (CABI 2021). It is one of the most difficult agricultural pests to control, causing losses in rapeseed of up to 50.0% (Finch 1989; Dosdall et al. 1994). Another related dipteran reported as a crucifer pest is *D. planipalpis* (Meigen), sister species of *D. radicum* and *D. floralis* (Fallén) (Dixon et al. 2014), whose main host is radish (*Raphanus sativus* L.) (Brooks 1951; Kelleher 1958). However, it has also been observed attacking commercial broccoli (Brassica oleracea L. var. italica Plenck), cabbage (B. oleracea var. capitata L.), cauliflower (B. oleracea var. botrytis L.), radish, turnip (*B. napus* L.), and wild crucifers such as field mustard (*B. campestris* L.), wild radish (*R. raphanistrum* L.), and London rocket (*Sisymbrium irio* L.) (Meraz-Álvarez et al. 2020). Nevertheless, most of studies have focused mainly on *D. radicum* and, to a lesser degree, on the onion fly (*D. antiqua* (Meigen)) (Finch 1989; Hemachandra et al. 2007). There are other species that are mainly saprophagous, such as *D. florilega* (Zetterdest) and *D. platura* (Stein), which have a wider range of hosts and can feed on decomposing tissue. However, under certain circumstances, they also feed on healthy tissues of plants of the family Brassicaceae and species of the genus *Allium* L., as well as legumes, Cucurbitaceae, and cereals (Griffiths 1993; Howard et al. 1994; Meraz-Álvarez et al. 2020).

Currently, there are few precedents of *D. planipalpis* and *D. platura* associated with crucifers in Mexico. Larvae of these dipterans cause damage mainly to the root crown of some cultivars of *B. oleracea*, and consequently, they affect the root system. Occasionally, the larvae develop near the broccoli head causing rot and malformation, or no formation of the inflorescence. In addition, in crops such as radish and turnip, direct damage caused by larvae to their edible part makes their commercialization difficult (Meraz-Álvarez et al. 2020). The presence of *D. planipalpis* and *D. platura* constitutes potential risk for production of this type of vegetable in Mexico, which in 2019 earned 1.113 billion dollars from export of broccoli, cabbage, and cauliflower, making Mexico the fourth largest producer worldwide (SIAP 2020). The production chain up to the end consumer requires much labor. Broccoli uses 76 workdays per season, compared with maize (the most cultivated crop in Mexico), which uses 17.25 workdays (Maldonado-Montalvo et al. 2017).

Because more than 60% of these crucifers are exported to international markets, compliance with strict sanitation, quality, and food safety regulation is required. These restrictions make it obligatory to use chemical control as one of the most common tactics for pest control (Bujanos et al. 2013). However, misuse of pesticides can lead to emergence of resistant populations, health problems for appliers, destruction of non-target arthropods (Pimentel et al. 1993; Lagunes-Tejeda et al. 2009), and residual pesticides in concentrations that are not acceptable in the destination countries, among other problems, and consequently, to loss of important markets. In this respect, *D. radicum* has developed resistance to chlorpyrifos in some areas where rutabaga (B. napus var. napobrassica (L.)) is grown in Canada (Blackshaw et al. 2012), and high concentrations of pesticide residues pollute aquifers (Joseph and Zarate 2015). In December 2007, the European Union banned chlorfenviphos, which was used to control *D. radicum* (Ferry et al. 2009); this, together with other restrictions that are being promoted in European countries to protect the environment and human health, presents another challenge for crucifer growers (Collier et al. 2020).

In addition, larvae of *Delia* are difficult to control because they are found in the soil or inside plant tissues, where it is difficult for sprayed insecticides to reach. For this reason, it is essential to identify natural enemies of these dipterans in crucifer-producing regions and contribute management options that use them as potential biological control agents. In this sense, the objective of this study was to search for and identify parasitoid insects and/or predators of *D. planipalpis* and *D. platura* in cultivated and wild crucifers.

## Methods

The study was conducted between February 2018 and February 2019 in Guanajuato, Puebla, and the State of Mexico, where some sites infested by *Delia* spp. were located (Table 1). The crops included in the collections were broccoli (Brassica oleracea var. italica Plenck), cabbage (B. oleracea var. capitata L.), turnip (*B. napus* L.), radish (*Raphanus sativus* L.), and other wild crucifers such as field mustard (*B. campestris* L.), London rocket (*Sisymbrium irio* L.), and wild radish (*R. raphanistrum* L.).

**Table 1.** Collection sites of samples infested by *Delia planipalpis* and *D. platura*.

Host	Site	
	Location	Date
*R. raphanistrum*	Colegio de Postgraduados Campus Montecillo, Texcoco, State of México 19°28'08.2"N, 98°54'04.7"W	19-II-2018
*S. irio*		29-III-2018
*R. sativus*		19-V-2018
*R. raphanistrum*		20-X-2018
*B. campestris*		19-XI-2018
*B. oleracea* var italica	San Diego de La Unión, Guanajuato 21°24'30.4"N, 100°45'19.3"W	25-X-2018
		04-XII-2018
*B. napus*	San Felipe Tenextepec, Tepeaca, Puebla 18°57'27.18"N, 97°50'50.24"W	04-IV-2018
*B. oleracea* var capitata		21-IX-2018
*R. raphanistrum*		08-XI-2018
*B. oleracea* var italica		08-XI-2018
*R. raphanistrum*	Los Reyes, Tepeaca, Puebla 19°00'01.1"N, 97°53'14.4"W	22-XI-2018
*R. sativus*		22-XI-2018
*B. oleracea* var capitata		22-XI-2018
*R. sativus*	Guadalupe Calderón, Tepeaca, Puebla 18°57'41.86"N, 97°50'32.44"W	06-XII-2018
*R. raphanistrum*		06-XII-2018
*B. oleracea* var italica	San Diego, Texcoco, State of México 19°30'09.8"N, 98°51'33.1"W	22-II-2019
*B. oleracea* var capitata		22-II-2019
		22-II-2019

Sampling was directed; 10 plants per site were selected, considering those that were observed to be stressed by water deficit and having wilting, yellowish, flaccid leaves and/or less vegetative development than the rest of the crop; these are characteristic symptoms of infestation by *Delia* spp. Wild crucifer plants were selected at random within and on the outer edges of commercial crops and the sample size varied from 5 to 10 plants depending on their abundance. Each plant was extracted with its root and adhered soil and placed in a polyethylene bag; additionally, with a post hole digger of 15.0 cm in diameter by 20.0 cm long, the soil of the first 10.0 cm of depth was collected taking as a reference point the exact site where the plant was extracted and because in this place the puparia are distributed due to the limited movement of third instar larvae around the plant and from the soil surface (Abu 1960). This soil was placed together with its respective plant in the polyethylene bag, which was labeled with the collection data.

The collected material was taken to the Entomology Laboratory at the Colegio de Postgraduados Campus Montecillo, Texcoco, Mexico, where the puparia and larvae were separated from the soil. A sieve with 2.0–2.8 mm openings was used to sift the soil and remove the *Delia* spp. larvae and puparia. To separate the larvae from damaged tissues, small cuts were made on the root and stem of the plants to search for galleries resulting from their feeding. These tissues were observed with an American Optical Model 570 stereoscopic microscope. The larvae obtained were placed in plastic 12.0 cm Petri dishes conditioned with moistened paper at the bottom on which two to three slices of radish were placed to provide them with food. In this way, they continued their development until reaching the pupal stage. The puparia obtained from sifting soil and those that resulted from collected larvae were observed under a microscope to separate *D. planipalpis* from *D. platura* using illustrations of Savage et al. (2016) as reference. The puparia were placed individually in 5.0 cm Petri dishes conditioned with moistened paper on the bottom and labeled with their collection data. This material was maintained in a rearing chamber at 26 ± 2 °C, 60 ± 10% relative humidity, and with a photoperiod of 12:12 (light: dark). Emergence of *D. planipalpis*, *D. platura*, and parasitoids was recorded daily. The emerged insects were collected, preserved in 70% alcohol in glass vials, and labeled with their respective data for later identification.

The keys of Campos and Sharkey (2006), Berry (2007), Forshage and Nordlander (2008), Quinlan (1978), and Nordlander (1981) were used to identify the parasitoids. Specimens were also sent for corroboration to the following specialists: Dr Juana María Coronado Blanco (Universidad Autónoma de Tamaulipas, specialist in the family Braconidae), Dr Fabiana Gallardo (School of Natural Sciences and Museum in Buenos Aires, Argentina, specialist in the family Figitidae), and Dr José Luis Navarrete Heredia (Universidad Autónoma de Guadalajara). The material is kept as reference specimens in the entomological collection of the Colegio de Postgraduados Campus Montecillo.

## Results

**Figure 1.**
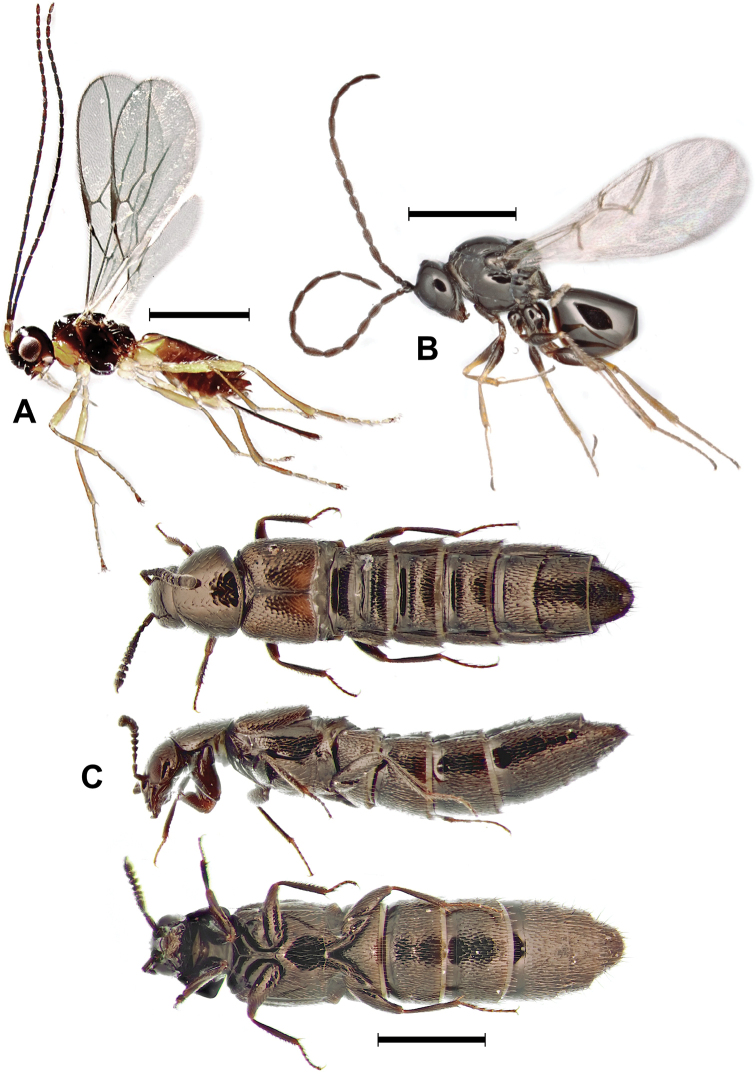
**A***Aphaereta pallipes* (Say) (Hymenoptera: Braconidae) **B***Trybliographa rapae* (Westwood) (Hymenoptera: Figitidae) **C***Aleochara bimaculata* Gravenhorst (Coleoptera: Staphylinidae). Scale bars: 1 mm.

A total of 321 *Delia planipalpis* and 49 *D. platura* puparia were collected in cultivated and wild crucifers in the states of Guanajuato, Puebla, and the State of Mexico (Table 2). From these puparia, three parasitoids emerged: *Aphaereta pallipes* Say (Hymenoptera, Braconidae), *Trybliographa rapae* (Westwood) (Hymenoptera, Figitidae), and *Aleochara bimaculata* Gravenhorst (Coleoptera, Staphylinidae) (Fig. 1). Of these three species, only in *A. pallipes* was gregarious habit observed, with up to 15 individuals obtained per *D. planipalpis* puparium.

**Table 2.** Parasitoids emerged from *Delia planipalpis* and *D. platura* puparia collected in commercial crops and weeds.

**Number of puparia collected**		**Emerged parasitoids**						**Host**
***D. planipalpis***	***D. platura***	***Aphaereta pallipes***		***Trybliographa rapae***		***Aleochara bimaculata***		
		♀♀	♂♂	♀♀	♂♂	♀♀	♂♂	
0	20	0	0	2	1	0	0	*R. raphanistrum*
0	14	0	0	2	11	0	0	*R. sativus*
11	2	0	0	0	0	1	0	*B. oleracea* var italica
18	0	7	4	0	0	0	0	
13	0	6	3	0	0	2	2	*B. oleracea* var capitata
57	8	56	15	0	0	0	0	*R. raphanistrum*
13	0	29	10	0	0	0	0	*B. oleracea* var italica
44	1	13	1	0	0	0	0	*R. raphanistrum*
34	2	22	7	0	0	0	0	*R. sativus*
53	2	29	19	0	0	0	0	*R. sativus*
44	0	93	39	0	0	0	0	*R. raphanistrum*

*Aphaeretha pallipes* emerged from puparia of *D. planipalpis* reared on B. oleracea var. italica, B. oleracea var. capitata, *R. sativus*, and *R. raphanistrum* from samples collected in Guanajuato and Puebla, *A. bimaculata* emerged from puparia of *D. planipalpis* and *D. platura* reared on B. oleracea var. italica and *capitata* from these places, and *T. rapae* emerged from puparia of *D. platura* reared on samples of *R. sativus* and *R. raphanistrum* from the State of Mexico.

## Discussion

The frequency of *Aphaereta pallipes* observed in the collected samples was 90%. In contrast, *Aleochara bimaculata* was present in 20% of the samples; likewise, the appearance of *Trybliographa rapae* was also minimal. Thus, *A. pallipes* is the most common parasitoid of *Delia planipalpis* in Guanajuato and Puebla.

Our findings contrast with what has been reported by other authors, who stated that *Aleochara bilineata* and *T. rapae* are the most common parasitoids of several root maggot species, including *D. planipalpis* and *D. platura* in Europe and Canada (Wilkes and Wishart 1953; Wishart 1957; Hemachandra et al. 2007), and *T. rapae* is capable of parasitizing up to 45% of their hosts (Wishart and Montheith 1954). In similar studies, additional species such as *Aphaereta difficilis* Nees, *A. tennuicornis* Nixon (Wishart et al. 1957), *A. auripes* (Provancher) (Wishart 1957), and the staphylinides *Aleochara bipustulata* and *A. verna* (Hemachandra et al. 2007) are named as occasional parasitoids of *Delia* spp. However, except for the presence of *T. rapae*, the species differ from our report.

Although *A. pallipes* has not been mentioned as an important parasitoid of *Delia* spp. in Europe and Canada, Tomlin et al. (1985) reported that *A. pallipes* and *A. bilineata* caused mortalities of up to 17 and 20%, respectively, in *D. antiqua* in commercial onion crops in southwestern Ontario. This braconid has a wide range of hosts and prefers members of at least three families, Coelophidae, Calliphoridae and Anthomyiidae (Wharton 1984), but it has also been reported in Sarcophagidae and Muscidae (Figg et al. 1983); therefore, the relative scarcity of *A. pallipes* may be due to its host preferences (Wilkes and Wishart 1953). Importantly, the presence of certain species and their percentages of parasitism are highly variable, depending on the crop, type of soil, geographic location (Wishart 1957), climate, host density (Jones and Hassell 1988; Turnock et al. 1995), differences in agronomic practices, habitat ecology of each crop, and even the number of host generations during the year (Nair and McEwen 1975).

In the case of *A. bimaculata*, our results are apparently different from those reported by other researchers in that most studies mention that *A. bimaculata* has been found parasitizing dipterans that develop in manure. Klimaszewski (1984) mentioned that *A. bimaculata* can be used as a biological control agent of some muscids, such as horn fly, *Haematobia irritans* L., and face fly, *Musca autumnalis* De Geer (Diptera, Muscidae). In addition, it has been reported that this staphylinid also attacks *Adia cinerella* Fallen (Diptera, Anthomyiidae) and *Scatophaga stercolaria* L. (Diptera, Scatophagidae) (Cervenka and Moon 1991), as well as *Musca domestica* L. (Diptera, Muscidae) (Wingo et al. 1967). Even though these are not insects of agricultural importance, the families to which they belong are closely related (Ding et al. 2015; Kutty et al. 2019). *A. bimaculata* has a Nearctic and Neotropical distribution (Maus et al. 1998) and that it is found in some regions of Mexico such as Guanajuato and Puebla (Navarrete-Heredia et al. 2002), it is expected and normal to find *A. bimaculata* parasitizing *Delia* spp. pupae in crucifer crops since species of *Aleochara* generally live in habitats where larvae of Cyclorrhapha dipterans develop (Maus et al. 1998).

Finally, although in this work no parasitoids were found in the wild crucifers *B. campestris* and *S. irio*, alternate hosts are important reservoirs of considerable populations of *Delia* spp. and consequently of their natural enemies, especially because they provide habitat during the season when there are no crops. In this way, wild crucifers assure that there is no scarcity of hosts for either the insect pest or their parasitoids and predators (Johnsen and Gutierrez 1997; Hemachandra 2007).

## Conclusions

Three species that emerged from puparia of *D. planipalpis* and *D. platura* collected in cultivated and wild crucifers were identified: one gregarious parasitoid, *Aphaereta pallipes* and two solitary parasitoids, *Trybliographa rapae* and *Aleochara bimaculata*. Only *Aphaereta pallipes* and *T. rapae* were specific to *D. planipalpis* and *D. platura*, respectively. Finally, *A. bimaculata* was associated with both pest species.

This is the first record of parasitoids of *D. planipalpis* and *D. platura* that occur naturally in Mexico. *Aphaereta pallipes* was the most abundant species, found in Guanajuato and Puebla, followed by *Aleochara bimaculata*, and finally *T. rapae*, which was found only in the State of Mexico. Knowledge of natural enemies of *Delia* spp. in crucifer-producing regions is only the starting point from which to broaden the search and begin to study their biological attributes with the aim of incorporating them into a biological control program against *D. planipalpis* and *D. platura*.
